# Slingshot homolog-1 expression is a poor prognostic factor of pT1 bladder urothelial carcinoma after transurethral resection

**DOI:** 10.1007/s00345-020-03092-4

**Published:** 2020-01-21

**Authors:** Qiang Luo, Yanxia Liu, Hu Zhao, Peng Guo, Qianwen Wang, Wenjun Li, Gang Li, Bin Wu

**Affiliations:** 1 Department of Urology, Jiangyin People’s Hospital, Affiliated Jiangyin Hospital of the Southeast University Medical College, No.163, Shoushan Rd, Jiangyin, 214400 Jiangsu Province China; 2 Department of Pathology, Jiangyin People’s Hospital, Affiliated Jiangyin Hospital of the Southeast University Medical College, Jiangyin, 214400 Jiangsu Province China; 3 Department of Urology, Tianjin Institute of Urology, the Second Hospital of Tianjin Medical University, Tianjin, 300211 China

**Keywords:** Slingshot homolog-1, pT1, Bladder urothelial carcinoma, Transurethral resection, Prognostic factor

## Abstract

**Objective:**

Slingshot homolog-1 (SSH-1) shows an important role in the occurrence and development in various tumors. While, the expression and prognostic implications of SSH-1 in bladder urothelial carcinoma (UC) remain unclear and thus were addressed in this study.

**Methods:**

Immunohistochemistry (IHC) was performed on tissue microarrays composed of 624 bladder UC specimens after transurethral resection of bladder tumor (TUR-BT) to detect SSH-1 expression. The clinic-pathological features were compared between SSH-1( +) and SSH-1(−) subgroups. The Kaplan–Meier curve with log-rank test and univariate/multivariate Cox regression model with stepwise backward elimination methods were performed for survival analyses.

**Results:**

In this study, 359 (57.53%) specimens were detected with SSH-1 expression. SSH-1 positivity was significantly associated with higher pathological grade (*p* = 0.020), lymphovascular invasion (*p* = 0.006), tumor recurrence (*p* < 0.001) and progression (*p* < 0.001) in bladder UC. Besides, SSH-1 positivity predicted a shorter overall survival (OS, *p* = 0.024), recurrence-free survival (RFS, *p* < 0.001), progression-free survival (PFS, *p* = 0.002) and cancer-specific survival (CSS, *p* = 0.047). Multivariate Cox proportional hazard analysis showed that tumor size (*p* = 0.007), lymphovascular invasion (*p* = 0.003), recurrence (*p* < 0.001), progression (*p* < 0.001) and SSH-1 expression (*p* = 0.015) were predictors of poor prognosis in bladder UC patients.

**Conclusions:**

SSH-1 expression was associated with undesirable clinic-pathological characteristics and poor post-operative prognosis in bladder UC patients. SSH-1 might play an important role in bladder UC and serve as a promising predictor of oncological outcomes in patients with bladder UC.

## Introduction

Malignant bladder tumor (BT) is the ninth commonest cancer worldwide, accounting for approximately 390,000 cases and 150,000 deaths per year [1]. However, BT is the seventh most common cancer in men and ranks thirteen according to mortality from all malignant tumors. The incidence of BT in North America, Europe, and Western Asia is higher, but mortality rate is greater in developing countries [2]. Urothelial carcinoma (UC), also known as transitional cell carcinoma, is the most common pathological subtype, which accounts for more than 90% of malignant BT [3]. Transurethral resection of bladder tumor (TUR-BT) has been the standard treatment for initial, non-muscle invasive bladder cancer (NMIBC), including carcinoma in situ (CIS), stage Ta and T1 BT [4].

Slingshot homolog-1 (SSH-1) is a kind of protein phosphatase, which could dephosphorylate and activate cofilin specifically. Cofilin is a F-actin-severing protein. SSH-1 conglutinates and co-localizes with F-actin, then the cofilin–phosphatase activity of SSH-1 increases obviously by adhering to F-actin [5, 6]. SSH-1 likely be a critical factor for stimulus-induced actin remodeling and vascular smooth muscle cell migration. SSH-1 has been found overexpression in various cancers, including pancreatic cancer [7], gastric cancer [8], colorectal cancer [9], etc. The expression of SSH-1 usually predicts a poor prognosis and survival in patients [10]. A comprehensive research about the relationship of SSH-1 expression and bladder UC patients has not been reported. In present study, we investigated the expression of SSH-1 in bladder UC with immunohistochemistry (IHC) and clinic-pathological characteristics with aims to explore whether SSH-1 could serve as a prognostic indicator of stage pT1 bladder UC.

## Patients and methods

### Patients

This study was approved by the ethics committee of Jiangyin People’s Hospital, and written informed consent was received from the subjects. A total of 624 bladder UC patients who underwent TUR-BT from Sep 2014 to Oct 2018 in our institution were enrolled in this study. No lymph node or distant metastasis was found through pre-operative examination in all patients. All resected specimens were reviewed by two experienced pathologists and diagnosed as stage pT1 bladder UC. The pathological stage was defined on the basis of the 2009 Union for International Cancer Control (UICC) TNM staging system, and tumor grading according to the 2004 WHO classification system for non-invasive urothelial system. All patients accepted intravesical chemotherapy regularly with epirubicin or gemcitabine and cystoscopy according to the EAU guidelines (2016) [11]. We did not use Bacillus Calmette–Guerin (BCG) because it has not been approved by China Food and Drug Administration before. The patients’ clinic-pathological information was obtained from medical records (Table 1). Formalin-fixed paraffin-embedded tissue blocks of post-operative tissues were collected for IHC examinations. All patients were divided into two cohorts according to the expression of SSH-1. To avoid the influence of tumor grade, we subsequently divided all cases into low-grade and high-grade subgroups with or without SSH-1 expression, respectively. Overall survival (OS), recurrence-free survival (RFS), progression-free survival (PFS) and cancer-specific survival (CSS) were performed for the analysis of prognostic implications. The PFS duration was calculated from first operation to the date when the disease developed to a higher pathological stage, histological grade and/or to lymphatic or distant metastasis.

**Table 1:** Clinic-pathological characteristics of the SSH-1( +) and SSH-1(−) patients with bladder urothelial carcinoma

Characteristics	SSH-1( +)	SSH-1(−)	*p* value
	*n* = 359 (57.53%)	*n* = 265 (42.47%)	
Mean age (years)	63.7 ± 15.5	65.8 ± 14.9	0.462
Sex			
Male	281 (78.27%)	198 (74.72%)	
Female	78 (21.73%)	67 (25.28%)	0.299
Tumor size (cm)			
< 3 cm	244 (67.97%)	186 (70.19%)	
≥ 3 cm	115 (32.03%)	79 (29.81%)	0.553
Pathological grade			
Low	156 (43.45%)	140 (52.83%)	
High	203 (56.55%)	125 (47.17%)	0.020
Tumor multiplicity			
Present	143 (39.83%)	95 (35.84%)	
Absent	216 (60.17%)	170 (64.16%)	0.311
Lymphovascular invasion			
Present	68 (18.94%)	29 (10.94%)	
Absent	291 (81.06%)	236 (89.06%)	0.006
Squamous differentiation			
Present	74 (20.61%)	43 (16.23%)	
Absent	285 (79.39%)	222 (83.77%)	0.165
Glandular differentiation			
Present	28 (7.80%)	16 (6.04%)	
Absent	331 (92.20%)	249 (93.96%)	0.396
Recurrence			
Present	202 (56.27%)	101 (38.11%)	
Absent	157 (43.73%)	164 (61.89%)	< 0.001
Progression			
Present	165 (45.96%)	76 (28.68%)	
Absent	194 (54.04%)	189 (71.32%)	< 0.001

### IHC and scoring

**Fig. 1:**
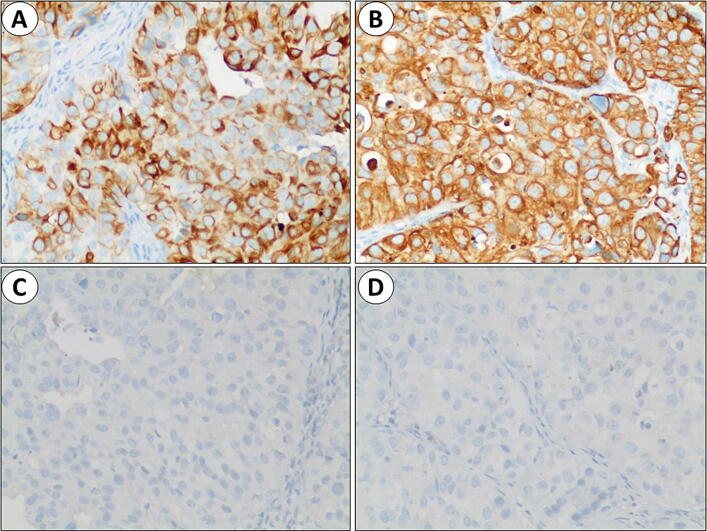
Immunohistochemistry staining of SSH-1 expression in bladder UC specimens.** a**,** b** positive expression of SSH-1 in cancer tissues; 400×magnification.** c**,** d** negative expression of SSH-1 in cancer tissues; 400×magnification

IHC staining was utilized to detect SSH-1 expression in the post-operative bladder UC tissues. Rabbit anti-SSH-1 antibody was purchased from Abcam. Co. (ab76943; Cambridge, UK) at a dilution of 1/1000, according to the protocol. Yellow or brown staining indicated SSH-1 positivity. The SSH-1 staining scoring system is based on published research [8]. The intensity of SSH-1 in cancer cells was graded as 0–3, and the number of SSH-1( +) cells was graded as 0–4 according to the percentage of positive cells (0: 0–5%; 1: 6–25%; 2: 26–50%; 3: 51–75%; 4: 76–100%). The sum of both grades defined as the final SSH-1 staining score (SSH-1( +): 4–7; SSH-1(−): 0–3) (Fig. 1).

### Statistical analysis

All statistical analysis was calculated with SPSS 22.0 (Chicago, IL, USA). The student’s* t* test and Chi-square test were performed to compare the clinic-pathological features between SSH-1( +) and SSH-1(−) subgroups. The survival outcomes were evaluated by Kaplan–Meier curves and differences were calculated by the log-rank test. Univariate and multivariate Cox proportional hazards models were used to evaluate prognostic factors. All tests were two sided, and a *p* value < 0.05 was considered to be statistically significant.

## Results

1. The association of SSH-1 expression in bladder UC specimens with clinic-pathological characteristics of included patients.
  The clinic-pathological characteristics of all 624 patients are shown in Table 1. SSH-1( +) was discovered in 359 (57.53%) cases of 624 bladder UC patients, SSH-1(−) was in 265(42.47%) cases. There was no statistical difference in mean age (*p* = 0.462), sex ratio (*p* = 0.299), tumor size (*p* = 0.553), tumor multiplicity (*p* = 0.311), squamous differentiation (*p* = 0.165) and glandular differentiation (*p* = 0.396) between SSH-1( +) and SSH-1(−) groups. While SSH-1 expression was significantly associated with higher pathological grade (56.55% vs. 47.17%, *p* = 0.020), lymphovascular invasion (18.94% vs. 10.94%, *p* = 0.006), and an increased occurrence of tumor recurrence (56.27% vs. 38.11%, *p* < 0.001) and progression (45.96% vs. 28.68%, *p* < 0.001). The low-grade bladder UC in SSH-1( +) and SSH-1(−) patients were 156 (43.45%) and 140 (52.83%) cases, and high-grade cases in two cohorts were 203 (56.55%) and 125 (47.17%), respectively.
2. The relationship between SSH-1 expression and patients’ survival outcomes.

**Fig. 2:**
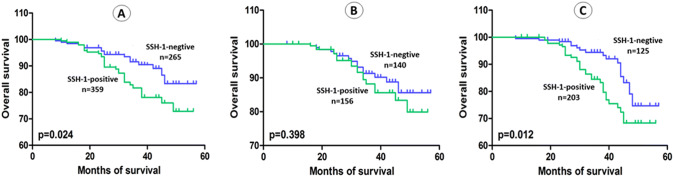
Kaplan–Meier curve of the overall survival (OS) for all patients (**a**), low-grade (**b**) and high-grade subgroups (**c**)

**Fig. 3:**
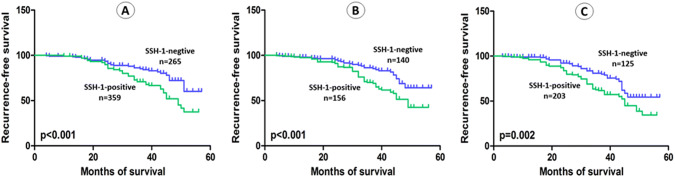
Kaplan–Meier curve of the recurrence-free survival (RFS) for all patients (**a**), low-grade (**b**) and high-grade subgroups (**c**)

**Fig. 4:**
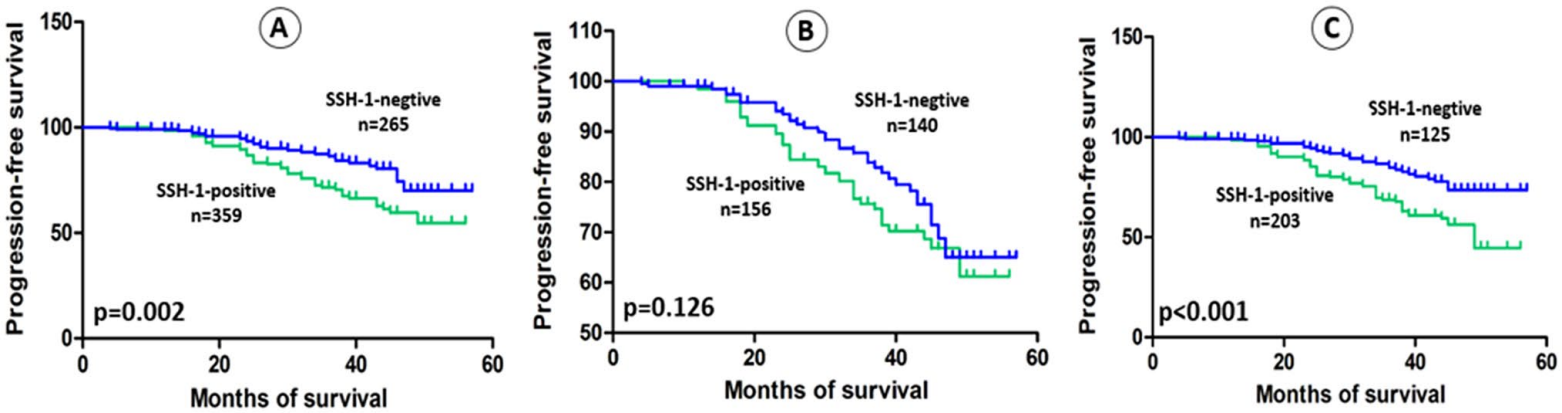
Kaplan–Meier curve of the progression-free survival (PFS) for all patients (**a**), low-grade (**b**) and high-grade subgroups (**c**)

**Fig. 5:**
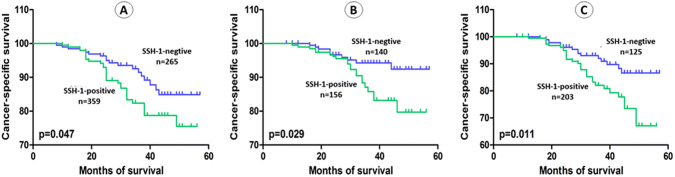
Kaplan–Meier curve of the cancer-specific survival (CSS) for all patients (**a**), low-grade (**b**) and high-grade subgroups (**c**)

The average follow-up in present study was 36.64 months (range: 3 to 57). Kaplan–Meier curve and log-rank test demonstrated that patients with SSH-1( +) had a shorter OS (*p* = 0.024), RFS (*p* < 0.001), PFS (*p* = 0.002) and CSS (*p* = 0.047) than patients of SSH-1(−) group (Figs. 2a, 3a, 4a and 5a). In subgroup of low-grade bladder UC, RFS (*p* < 0.001) and CSS (*p* = 0.029) were also shorter in SSH-1( +) patients (Figs. 3b, 5b). However, no statistical difference was found in PFS (*p* = 0.126) and OS (*p* = 0.398) between the two subgroups (Figs. 2b, 4b). In subgroup of high-grade bladder UC, SSH-1 positivity was significantly associated with poor OS (*p* = 0.012), RFS (*p* = 0.002), PFS (*p* < 0.001) and CSS (*p* = 0.011) than SSH-1 negativity patients (Figs. 2c, 3c, 4c and 5c).

The univariate analysis model indicated that tumor size (HR = 1.566, *p* = 0.015), pathological grade (HR = 1.325, *p* = 0.024), lymphovascular invasion (HR = 1.838, *p* < 0.001), squamous differentiation (HR = 1.492, *p* = 0.032), tumor recurrence (HR = 3.451, *p* < 0.001), progression (HR = 2.947, *p* < 0.001), and SSH-1 positive expression (HR = 1.693, *p* < 0.001) showed prognostic implication for the predication of CCS in patients with bladder UC (Table 2). In the multivariate Cox regression model, tumor size (HR = 1.429, *p* = 0.007), lymphovascular invasion (HR = 1.692, *p* = 0.003), recurrence (HR = 3.856, *p* < 0.001), progression (HR = 2.655, *p* < 0.001) and SSH-1 positive expression (HR = 1.558, *p* = 0.015) were significant variables of CSS (Table 2). The results indicated that SSH-1 expression is a specific predictor of poor CSS for stage pT1 bladder UC patients.

**Table 2:** Univariate and multivariate Cox regression analyses of cancer-specific survival of patients with bladder urothelial carcinoma

Variables	Univariate analysis			Multivariate analysis		
	HR	95% CI	*p* value	HR	95% CI	*p* value
Age (years)	1.031	0.894–1.220	0.463			
Sex	1.108	0.912–1.365	0.574			
Tumor size	1.566	1.206–2.118	0.015	1.429	1.283–1.804	0.007
Pathological grade	1.325	1.186–1.660	0.024	1.213	0.944–1.589	0.147
Tumor multiplicity	1.291	0.847–1.693	0.282			
Lymphovascular invasion	1.838	1.337–2.506	< 0.001	1.692	1.360–2.054	0.003
Squamous differentiation	1.492	1.255–1.906	0.032	1.220	0.921–1.759	0.188
Glandular differentiation	1.190	0.751–1.543	0.238			
Recurrence	3.451	2.683–5.770	< 0.001	3.856	3.007–5.402	< 0.001
Progression	2.947	2.380–4.136	< 0.001	2.655	2.103–3.458	< 0.001
SSH-1 expression	1.693	1.235–2.106	< 0.001	1.558	1.192–2.043	0.015

## Discussions

Bladder UC is the most common pathological subtype of malignant BT. Approximately 75% of bladder UC patients presented with non-muscle-invasion bladder cancer (NMIBC), including carcinoma in situ (CIS, 10%), stage Ta (mucosa, 70%) and T1 (submucosal invasive, 20%). NMIBC is usually managed with complete TUR-BT and post-operative intravesical chemotherapy [12]. The surgery quality of initial TUR-BT directly influences the accurate diagnosis, pathological stage, adjuvant therapy, and survival prognosis of NMIBC. For NMIBC, some reports have showed that the 5-year recurrence rate after TUR-BT ranged from 50–70%, and incomplete resection was considered the major reason [13]. The quality control indicators of TUR-BT include complete resection of visible tumors, second-look TUR-BT (or be called re-TUR-BT) for stage pT1 and high-grade BT, adjuvant intravesical chemotherapy or immunotherapy with BCG, presence of detrusor muscle in the excised tissues and no bladder perforation [14, 15]. Although second-look TUR-BT has been recommended for patients with stage pT1 BT after initial operation for re-staging and deciding the subsequent therapy. Some scholars queried the need for such additional surgical intervention, considering the patients and healthcare burdens, and the survival benefits of second-look TUR-BT is still controversial at present [12, 16]. Hence, the majority of patients with stage pT1 bladder UC were not performed re-TUR-BT in our institution. To avoid the selection bias of included cases, patients who accepted re-TUR-BT after initial surgery were excluded in this study.

The SSH family protein phosphatases contain a highly conserved non-catalytic region (also be called “SSH-N domain”), which closes to the N-terminus of phosphatase catalytic domain [17, 18]. The N-terminal half of SSH-1 (amino acids 1–461) has the ability of cofilin–phosphatase and F-actin-mediated activation. This reminded us that SSH-1 might play an important role in determining the substrate specificity of SSH-1 cytokine and in mediating F-actin-mediated activation of the cofilin–phosphatase [19]. Some evidences have confirmed that the activation of cofilin was critical for membrane protrusion formation, directional cell migration, and cancer cell invasion. Cofilin phosphoregulation by SSH-1 is a pivotal factor of actin cytoskeletal remodeling and involving in cancerous cells migration and metastasis [20]. Aggelou et al. [9] discovered that LIM kinases 1, LIM kinases 2 and SSH-1 were regulators of actin dynamics cofilin and contributed to colorectal cancer progression and chemoresistance to 5-fluorouracil. These cytokines were also associated with epithelial–mesenchyma transformation (EMT) markers, such as downregulation of E-cadherin and positive expression of ZEB. Wang et al. [7]found that the expression of SSH-1 was to be upregulated in pancreatic cancer cell lines with high metastatic potential, and loss of SSH-1 was correlated with an increase in the phosphorylation of cofilin-1 and the inhibition of cancerous cell migration (but not proliferation). Moreover, SSH-1 was also significantly associated with lymph node metastasis in gastric cancer patients, and was an independent predictor of poor clinical outcomes in Maimaiti’s research [8]. While, little is known regarding the SSH-1 expression in human stage pT1 bladder UC patients.

In present study, we focused on the prognostic significance of SSH-1 expression in stage pT1 bladder UC because patients with this disease after TUR-BT generally suffer from tumor recurrence and progression to invasive cancers [11, 15]. A total of 624 bladder UC tissues were brought into this study, and 359 (57.53%) cases showed SSH-1 positivity. The presence of SSH-1 in bladder UC specimens was significantly associated with unfavorable clinic-pathological characteristics, including higher pathological grade, lymphovascular invasion, and incidence of tumor recurrence and progression. Through follow-up and survival analysis, we identified that bladder UC patients with SSH-1 expression had a significantly poor OS, RFS, PFS, and CSS. We subsequently divided these patients into low-grade and high-grade subgroups on the basis of pathological examination. In the subgroup analysis, SSH-1 positivity patients were still statistically associated with poorer oncological outcomes than SSH-1 negativity subgroup. Only in the PFS and OS of low-grade bladder UC patients, no significant difference was discovered between the two subgroups. Several predictors or prognostic markers for tumor recurrence, progression or cancer-specific death of bladder UC patients had been reported in previous studies, such as sex, age, tumor size, tumor multiplicity, pathological grade, CIS, T1 substaging, histologic features and Ki-67 proliferation index [21–23]. Additionally, some scholars pointed that the presence of squamous and/or glandular differentiation was correlated with a higher recurrence rate and poor survival in NMIBC patients, such cellular heterogeneity could also be an independent prognostic predictor of bladder cancer [24, 25]. Nevertheless, Kim et al. [26] revealed the survival outcomes of bladder UC patients with squamous and/or glandular differentiation were similar to patients with pure bladder UC, given comparable demographic, clinic-pathologic and management features. Bladder UC with differentiation just presented with higher pathologic stage. Similar results had been reported in other literatures as well [27, 28]. In this study, the multivariate Cox regression analysis showed that tumor size, lymphovascular invasion, recurrence, progression, and SSH-1 positivity were prognostic indicators influencing the CSS of stage pT1 bladder UC patients. Other variables, including pathological grade, tumor multiplicity, squamous and glandular differentiation had no significant impacts on the CSS of patients.

There are three limitations in our study. First, this is a retrospective research and inherent biases were unavoidable in patients’ selection and treatment. Second, we did not recruit a large number of patients from multiple medical centers, and some patients in this study were not followed up for a long time. Third, we did not perform the molecular experiments and explore the mechanisms of this phenotype.

## Conclusions

This study first concentrated on the oncologic outcomes of SSH-1 expression in stage pT1 bladder UC patients. We clarified the clinic-pathological significance of SSH-1 expression in such patients and discovered that SSH-1 was significantly associated with an increased risk of tumor recurrence and progression. SSH-1 might be an independent predictor and promising biomarker for the treatment and/or progression of pT1 bladder UC.

## Data Availability

The data and materials of this study are available from the corresponding author upon reasonable request.

## Abbreviations

SSH-1: Slingshot homolog-1
UC: Urothelial carcinoma
IHC: Immunohistochemistry
TUR-BT: Transurethral resection of bladder tumor
OS: Overall survival
RFS: Recurrence-free survival
PFS: Progression-free survival
CSS: Cancer-specific survival
BT: Bladder tumor
NMIBC: Non-muscle invasive bladder cancer
CIS: Carcinoma in situ
UICC: Union for International Cancer Control
BCG: Bacillus Calmette–Guerin
EMT: Epithelial–mesenchyma transformation
